# Editorial: Coaching female athletes

**DOI:** 10.3389/fspor.2025.1690341

**Published:** 2025-10-28

**Authors:** Khatija Bahdur, Tihana Nemčić Bojić, Fraser Carson

**Affiliations:** ^1^ Department of Sport, LUNEX, Differdange, Luxembourg; ^2^ Luxembourg Health and Sport Sciences Research Institute A.S.B.L., Differdange, Luxembourg; ^3^ Faculty of Kinesiology, University of Zagreb, Zagreb, Croatia

**Keywords:** health and physiology, performance optimization and monitoring, coaching, leadership and support systems, athlete well-being and long-term development

**Editorial on the Research Topic**
Coaching female athletes

The number of female athletes participating at high-performance levels is increasing. This has created more opportunities for competition in professional leagues and raised the standards of national team competitions in numerous sporting codes (1). However, a disproportionate volume of sports research tends to focus overtly on men, and these results are applied (often incorrectly) to female athletes (2). Biological, psychological, emotional, and social differences exist, and when combined with the environmental, infrastructural, and resource differences within female sports, there is a need to create female-specific knowledge for coaches.

Contemporary research suggests that many studies within the domain of female athletes were conducted with a gendered bias and used research to reinforce existing gender norms. There are emerging critiques of existing sports science literature that tend to reinforce traditional social constructs, with female athletes seen as fragile, in need of care, and nurturing. The concept of women being unable to accept criticism or being too emotional or fragile, physically not being aggressive enough, and not being able to handle coaching methodologies that are usually associated with men's teams has resulted in the conclusion that sometimes coaches in women's sports “hold back”. Female athletes are also often referred to as different, and coaching them is considered challenging (3). These negative stereotypes, in fact, are likely to hinder the progress of female athletes (4).

As women's sports grow, structural and cultural shifts occur, which may result in changing expectations (5). Many previous studies were conducted in an environment in which, regardless of participation level, pathways to professionalism for female athletes were minimal. Even within elite female sports, athletes often lack access to the same resources and developmental background as their elite male counterparts (6). Currently, this is changing in some countries and sports. Despite higher competition levels and more women pursuing high-level sports opportunities, a persistent societal misconception remains that women in sports are primarily driven by social engagement rather than competition and performance (5). It remains to be seen how the expectations and requirements for the now increasing number of professional female players will impact coaching methodologies (7).

In this Research topic, we focused on “*Coaching female athletes*”, with 12 published research articles that fit within the broader themes of i) Health and Physiology, ii) Performance Optimization and Monitoring, iii) Coaching, Leadership, and Support Systems, and iv) Athlete Well-being and Long-Term Development. Within the health and physiology theme, Jones et al. highlighted the complexities associated with female reproductive health and performance in a longitudinal study of elite British track and field athletes. These athletes perceived an inferior performance when estrogen and progesterone levels were low. The authors advocated for more support for athletes and individual strategies to be utilized to optimize athlete health and performance. Roffler et al. noted that menstrual cycle symptoms vary widely within the same team, and that tracking symptoms can inform an individualized healthcare and management approach that can be used to help coaches adjust training load. Meanwhile, Vardardottir et al. demonstrated that repeated patterns of low energy availability and low carbohydrate intake increase the risk of relative energy deficiency (REDs) for female athletes.

Regarding the performance optimization and monitoring theme, which aims to help coaches and sports scientists predict performance development in young female athletes, Romann et al. focused on the development of a new analysis method to improve reliability and provide a predictive model for future performance. This model can help coaches individualize training programs. Stojiljković et al. created a valid and reliable tool to assess drop jump performance interlimb asymmetry in young female basketball players. The My Jump 2 app is low-cost and easy for coaches to use to improve and monitor jumping performance. Sammoud et al. hypothesized that backward running as a training method would provide greater physical fitness benefits than forward running for young female handball players. Their findings suggest that improved performance may come from a combination of both. Adding important information to help profile the physical performance attributes of elite female handball players, an area that has been largely overlooked in the literature, the results by Radovic et al. related to eccentric performance characteristics can be used by coaches to guide structured training programs focused on lower-body strength and power.

Focusing on coaching, leadership, and support systems, Mashilo and Kubayi investigated the coach-athlete dyadic relationship for South African school athletes and the importance of trust within the partnership. They concluded that coaches need to promote fairness in all aspects of their coaching and individualize encouragement and support. In our own contribution (Carson et al.), we explored the intense tournament environment and how a coach navigates the additional pressures and time constraints to build a team. Individual feedback sessions and empowering the female athletes were important for the coach and instrumental in building a trusting relationship. In their study, Alruwaili focused on how to increase Saudi Arabian female participation in sports and identified that a transformational leadership style, with an emphasis on inspirational motivation, is an effective means to engage more women in physical activity and break down sociocultural barriers.

The final two studies focused on athlete well-being and long-term development. Zach et al. addressed the difficult topic of sexual harassment among athletes and highlighted the differences in perceptions and interpretations of sexual harassment. The authors reinforce the need for improved education and awareness to promote a safer culture in sports and encourage sports organizations to create better-defined policies and adopt a zero-tolerance approach. Ehnold et al. investigated engagement with off-field educational programs for female soccer players in Germany. Despite the growing professional opportunities for female soccer players, over 90% of the players were actively engaged in academic or vocational education. It is known that commitment to other activities outside the sporting environment can help athletes develop different identities, which is important for long-term development.

The 12 manuscripts in this research topic provide valuable insights into the developing literature on coaching female athletes. Interestingly, there appear to be few differences in the barriers that are faced by women who wish to participate in sports worldwide. Despite increased attention to women's sports, access to facilities, resources, and quality support remains limited. From a coaching perspective, female athletes differ from male athletes, and we are only just beginning to build the research base to show how women can be coached most effectively. An athlete-centered approach to coaching is crucial, with a positive coach-athlete relationship appearing more important for female athletes than male athletes. As such, coaches are encouraged to develop their relational skills and ensure individualization for each athlete they engage with. This individualization is important for all areas of female athlete performance, including training load, athletic development, support, health, and well-being.
